# A bisphosphonate for ^19^F-magnetic resonance imaging

**DOI:** 10.1016/j.jfluchem.2016.02.008

**Published:** 2016-04

**Authors:** Gavin D. Kenny, Karen P. Shaw, Saranja Sivachelvam, Andrew J.P. White, Rene M. Botnar, Rafael T.M. de Rosales

**Affiliations:** a Division of Imaging Sciences & Biomedical Engineering, King’s College London, St Thomas’ Hospital, London SE1 7EH, UK; b Department of Chemistry, Imperial College London, Exhibition Road, South Kensington, London SW7 2AZ, UK

**Keywords:** ^19^F-MRI, Bisphosphonates (BPs), Magnetic resonance imaging (MRI), Preclinical imaging, Fluorinated bisphosphonate

## Abstract

^19^F-magnetic resonance imaging (MRI) is a promising technique that may allow us to measure the concentration of exogenous fluorinated imaging probes quantitatively *in vivo.* Here, we describe the synthesis and characterisation of a novel geminal bisphosphonate (^19^F-BP) that contains chemically-equivalent fluorine atoms that show a single and narrow ^19^F resonance and a bisphosphonate group that may be used for labelling inorganic materials based in calcium phosphates and metal oxides. The potential of ^19^F-BP to provide contrast was analysed *in vitro* and *in vivo* using ^19^F-MRI*. In vitro* studies demonstrated the potential of ^19^F-BP as an MRI contrast agent in the millimolar concentration range with signal-to-noise ratios (SNR) comparable to previously reported fluorinated probes. The preliminary *in vivo* MRI study reported here allowed us to visualise the biodistribution of ^19^F-BP, showing uptake in the liver and in the bladder/urinary system areas. However, bone uptake was not observed. In addition, ^19^F-BP showed undesirable toxicity effects in mice that prevent further studies with this compound at the required concentrations for MRI contrast. This study highlights the importance of developing ^19^F MRI probes with the highest signal intensity achievable.

## Highlights

• A trifluoromethyl-bisphosphonate for ^19^F-MRI was synthesised and characterised.
• *In vitro* studies showed its potential with properties comparable to previous probes.
• *In vivo* ^19^F/^1^H-MRI studies showed fast renal excretion and liver uptake.

## Graphical abstract

A water-soluble 1,1-geminal fluorinated bisphosphonate showing a single and narrow ^19^F resonance was synthesised and characterised. Its properties as contrast agent for ^19^F-MRI were evaluated *in vitro* and *in vivo.*

**Figure:**
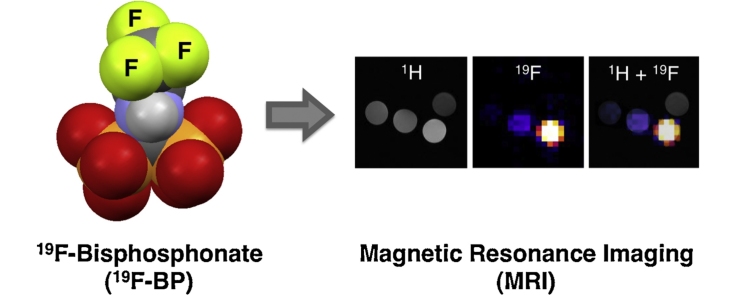

## 1 Introduction

MRI is a medical imaging technique that offers high-resolution images of soft tissues without the need for ionising radiation. In addition, and unlike other techniques such as those based on radionuclides, it does not require the injection of contrast agents in order to obtain meaningful images. However, for some imaging procedures such as angiography or molecular imaging, chemical compounds can be used to enhance the contrast of the specific tissue of interest. In this context, one area that MRI currently lags behind other imaging modalities, particularly positron emission tomography (PET) and single photon emission computed tomography (SPECT), is the quantitative measurement of the signal provided by these contrast agents. This is a key requirement for molecular imaging applications. Current contrast-based MR techniques rely on the detection of imaging agents containing paramagnetic ions such as gadolinium, manganese or iron. However, interpretation of the results is difficult due to the varying underlying signal hyper- and hypo-intensities in MRI. In answer to this ^19^F-MRI has been implemented. The use of fluorine as the nucleus for magnetic resonance has several advantages over protons. First, the lack of endogenous MR-visible fluorine provides an unambiguous readout of the introduced fluorine-containing compounds location. In addition the ^19^F MR signal can be quantified, giving a measure of the contrast agent’s concentration. This is in contrast to paramagnetic contrast agents used in ^1^H-MRI and based on Gd, Mn and particularly Fe, where *in vivo* absolute quantification is not achievable.

The main uses of ^19^F-MRI in biomedical imaging to date has been for cell tracking [1], [2], [3], [4], [5] visualisation of inflammation [6], [7], [8], [9] and for imaging angiogenesis [10], [11] all using ^19^F nanoparticles. This is an obvious choice due to the capacity of nanoparticles to carry the many fluorine atoms required to obtain sufficient signal. More recently attempts have been made to image smaller compounds by modulating the ^19^F signal using lanthanide metals [12], [13] and used for the detection of gene expression [14]. Despite these early promising results and clear advantages for molecular imaging compared to ^1^H-MRI, ^19^F-MRI remains underused in clinical practice. This is due to a major disadvantage, which is low sensitivity [15]. As a consequence most ^19^F-MRI probes designed to date need to have many fluorine atoms to provide enough signal in the tissues of interest (∼20–50 mM ^19^F). However, the number of fluorine atoms that a molecule can carry is limited for several reasons. First is solubility, as the fluorine content of a molecule increases, the water solubility decreases. The second limitation is the number of ^19^F signals, the ideal ^19^F-MRI contrast agent having one single narrow resonance to maximise signal and avoid imaging artifacts. To achieve this all the fluorine atoms must be in the same chemical and magnetic environment. Another limitation of ^19^F-MRI is related to the long longitudinal relaxation times (*T*_1_) of the fluorine nucleus (∼1–2 s). This translates into long acquisition times for the MRI procedure due to the 5–10 s required between radiofrequency (RF) pulses, which results in long times or more complex non-standard MRI sequences.

**Scheme 1:**
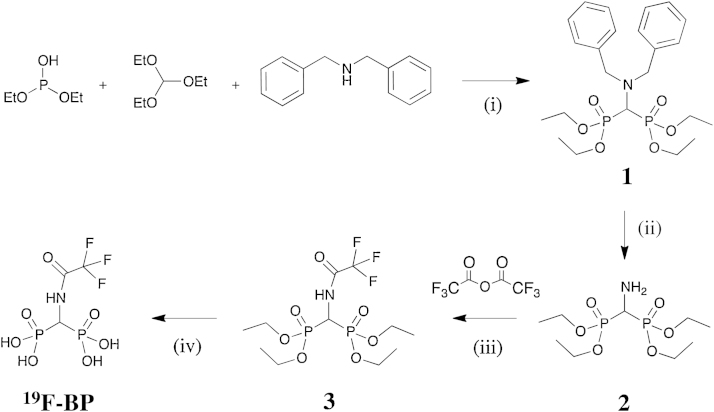
The synthetic scheme of ^19^F-BP. (i) 29 h at 150–160 °C; (ii) H_2,_ 10% Pd/C catalyst in EtOH, room temperature; (iii) 3 h in dry DCM; (iv) (a) 24 h, Me_3_SiBr (15 eq) in dry DCM, room temperature (b) 1.5 h MeOH, 1.5 mL, room temperature.

We are interested in developing ^19^F-MRI contrast agents for molecular imaging that show single and narrow ^19^F resonances and short *T*_1_ relaxation times. Previously we have shown that 1,1-bisphosphonates (BPs) bind very strongly to metabolically active bone and calcium phosphate materials such as hydroxyapatite using SPECT and PET imaging [16], [17], [18], [19]. In addition, we found that BPs also bind very strongly to many nanomaterials based on lanthanide metal oxides of the type M_2_O_3_ (M = Gd, Er, among others) with known relaxation rate-enhancement properties [19]. We hypothesised that a fluorinated BP molecule could be an useful tool in the development of ^19^F-MRI probes, that would allow to combine of the amplification properties of nanoparticle-based platforms (high numbers of equivalent fluorine atoms) with the relaxation-enhancement properties of lanthanide-based materials (short acquisition times) without affecting their water solubility. In this way we could potentially achieve ^19^F-MRI probes with high signal intensity and sensitivity that could be imaged in a short time. In addition, their solution and *in vivo* properties could be easily controlled by surface modification using the same BP chemistry. In this work, we report our first attempts at achieving this aim by synthesizing and characterising a new fluorinated BP (**^19^F-BP**, Scheme 1) and evaluate for the first time its properties as a single molecule for ^19^F-MRI *in vitro* and *in vivo.*

## 2 Results and discussion

### 2.1 Synthesis

**Fig. 1:**
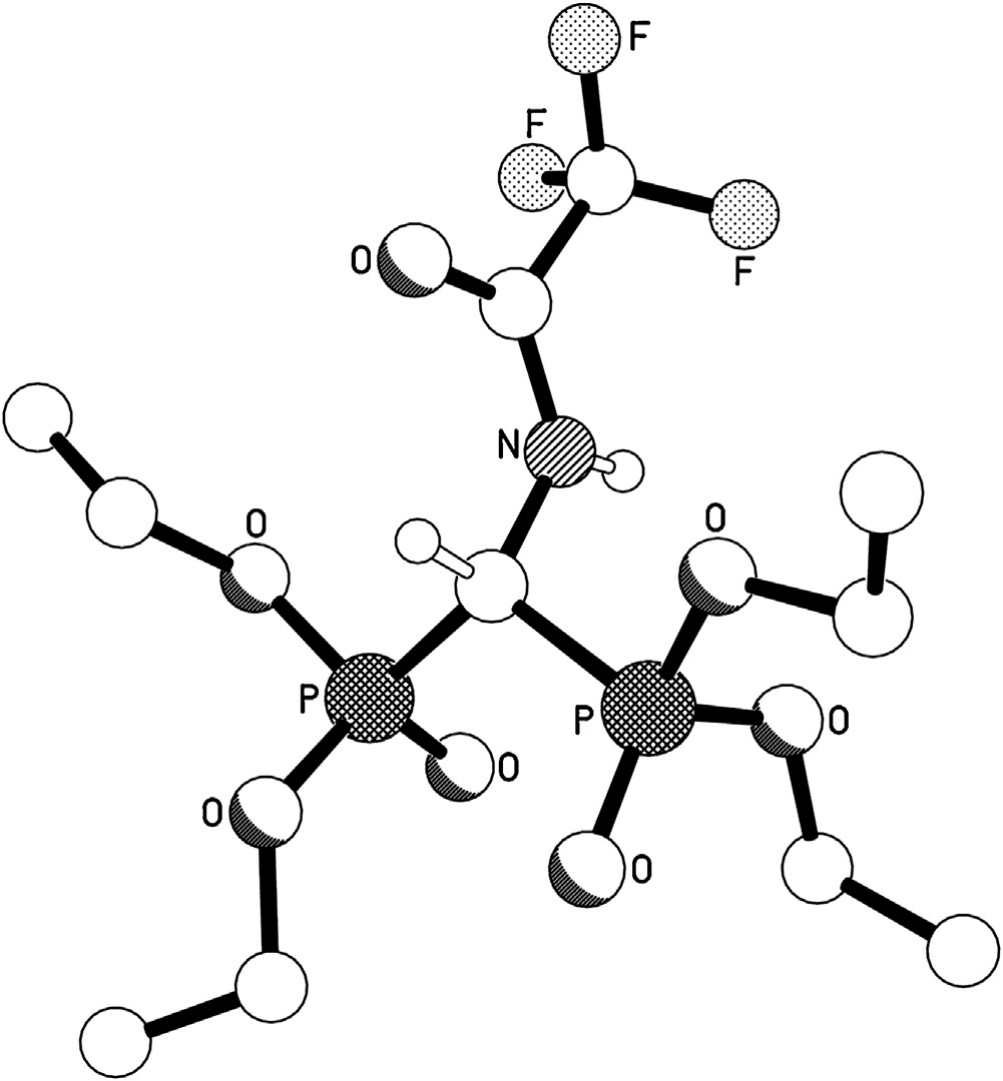
The molecular structure of **3** (CCDC ID 960161).

The reaction scheme for the synthesis of **^19^F-BP** is shown in Scheme 1. Tetraethyl aminomethyl-bisphosphonate (**2**) was synthesized following published methods [20], [21]. Briefly, diethyl phosphite, triethylorthoformate and dibenzylamine were reacted for 29 h at 150–160 °C to yield the benzylated bisphosphonate (**1**). The amino group of **1** was deprotected with H_2_ and 10% Pd/C catalyst to yield **2**. After removal of the catalyst, **2** was reacted with 2.9 equivalents of trifluoroacetic anhydride (TFAA) in dry DCM for 3 h. Excess TFAA was used in order to prevent low reaction yields due to potential hydrolysis of the anhydride. After evaporation of the volatiles and work-up, **3** was recrystallised from cold hexanes in good yields (78%). The compound was characterised by NMR, HR-MS and the structure confirmed by X-ray crystallography (Fig. 1 and Fig. SI)

The ethyl-protected bisphosphonate group of **3** was deprotected by reacting with excess bromotrimethylsilane followed by methanolisis at room temperature. The reaction gave quantitative yields of **^19^F-BP** as assessed by NMR and MS, confirming complete removal of the ethyl protecting groups. ^19^F-NMR and ^31^P-NMR also confirmed the stability of the trifluoromethyl and bisphosphonic groups, respectively. The solubility properties of **3** changed from hydrophobic to hydrophilic after deprotection, as expected for bisphosphonic acids, and allowed us to perform our imaging studies in water. One of the main advantages of this compound over most ^19^F-MRI contrast agents reported to date based on perfluorinated molecules is the chemical equivalence of its F atoms. Non-equivalent F atoms result in broad and/or multiple resonances that have a negative effect on the final ^19^F-MRI signal. In **^19^F-BP**, however, having a narrow single ^19^F resonance (−76.15 ppm, *ω*_1/2_ = 4.9 Hz), maximises imaging signal and minimises the appearance of image artefacts.

### 2.2 *In vitro* MR imaging studies

**Fig. 2:**
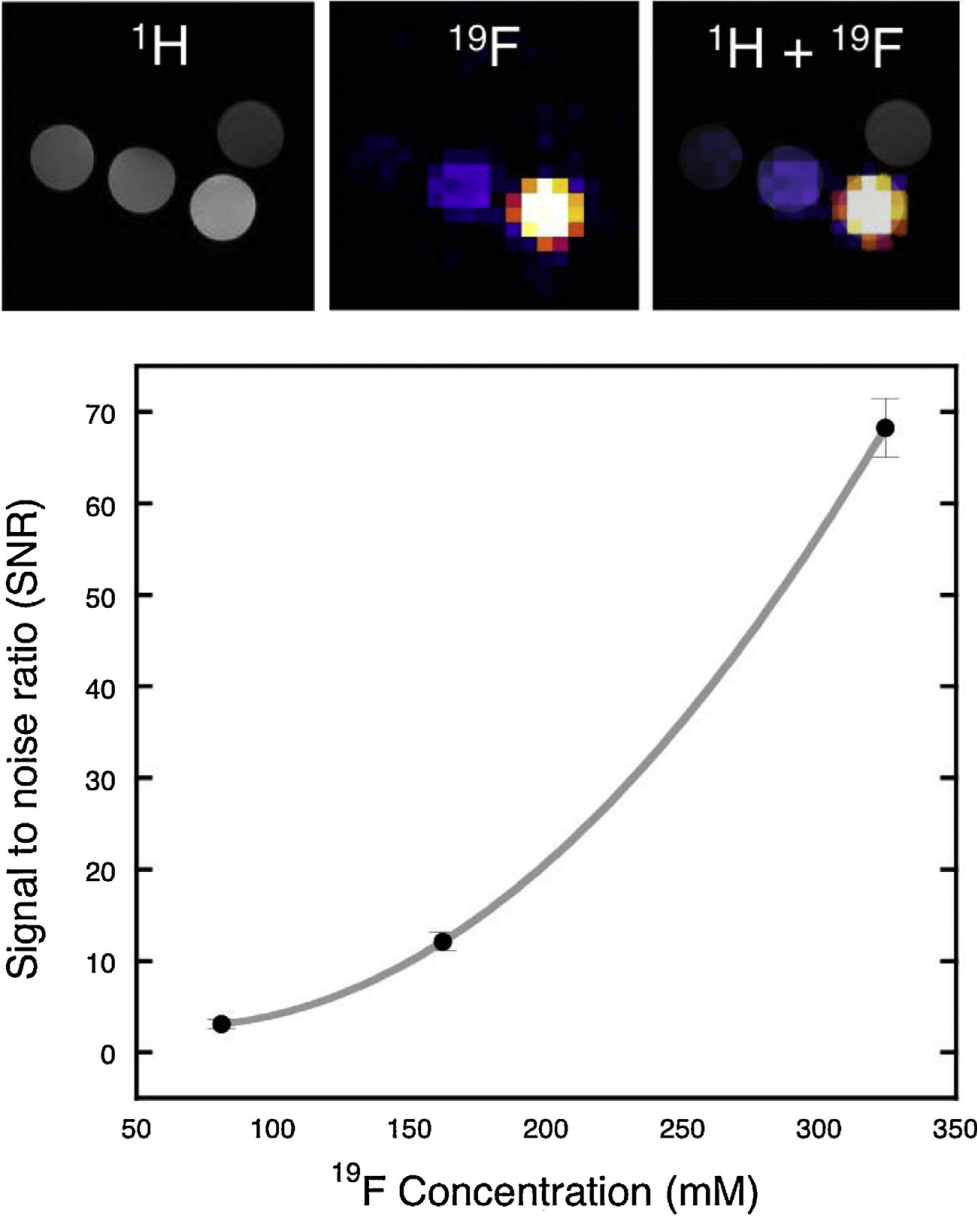
*In vitro* MR imaging study. Top: ^1^H and ^19^F MRI phantom study with vials of ^19^F-BP at increasing concentrations (27, 54 and 108 mM) in water from left to right, with water above. Bottom: graph showing the increase of signal to noise ratio (SNR) with increasing probe concentration. The fit is a smooth curve fit intended to represent the trend. Error bars are the result of 3 ROI image analyses.

Phantom MRI studies were performed to evaluate the contrast properties of ^19^F-BP (Fig. 2). The compound was dissolved in water at pH 7 at several concentrations (27, 54 and 108 mM) and imaged in a preclinical 9.4 T MRI scanner. A clear concentration-dependent increase in signal intensity and signal to noise ratio (SNR) was found, demonstrating that **^19^F-BP** can be imaged in the high mM concentration range. Stability studies were also performed using these samples. The ^1^H NMR and ^19^F-MRI spectra remained stable for 5 h at pH 7 and 37 °C, confirming the stability of **^19^F-BP** at these conditions. This gave us confidence to study its biodistribution properties *in vivo*.

### 2.3 *In vivo* MR imaging studies

**Fig. 3:**
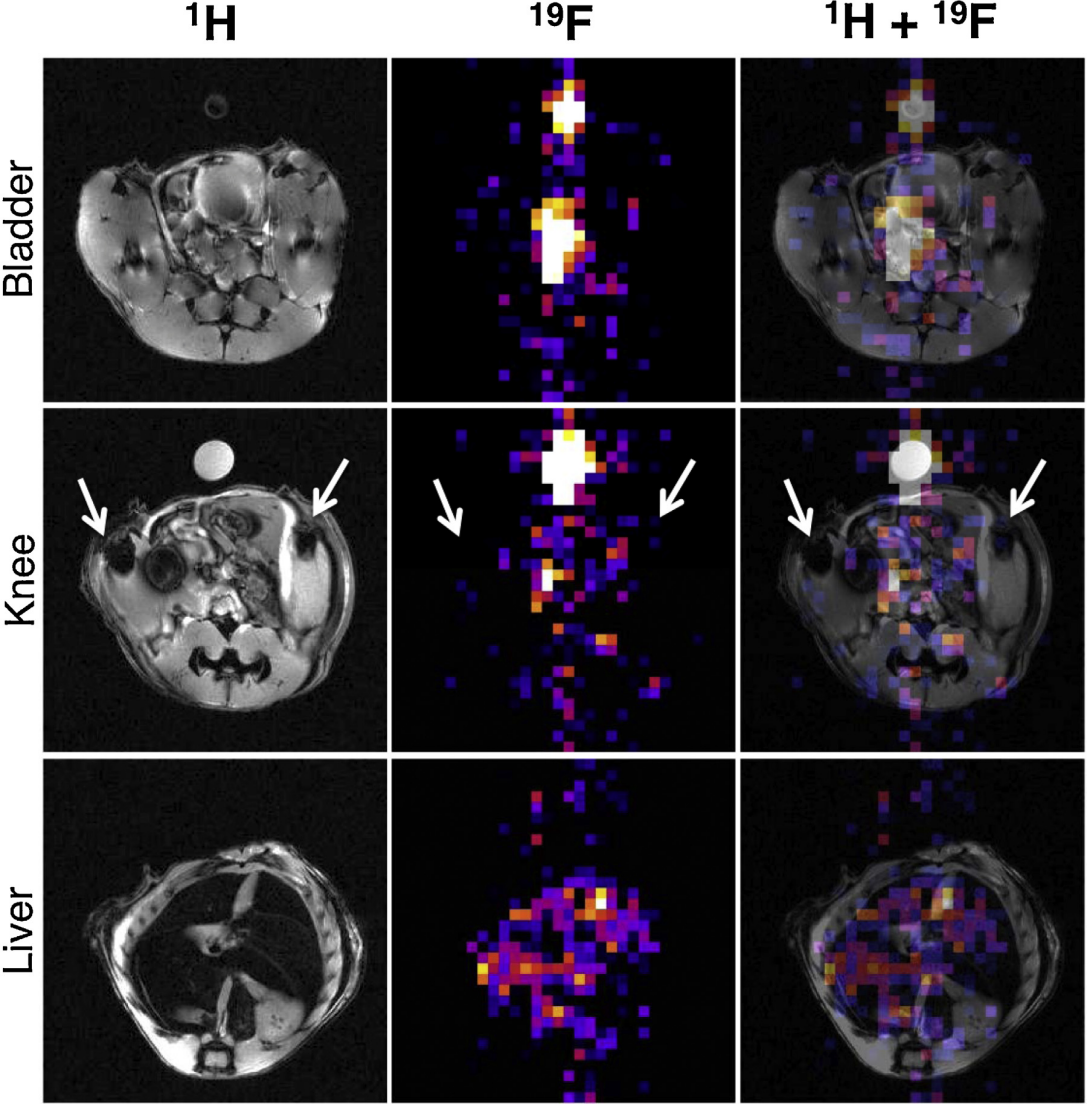
Animal MR imaging study (see Section 4 for details). A mouse was injected with ^19^F-BP i.v. (108 mM in PBS buffer) and imaged using ^1^H (left column) and ^19^F MRI (middle column), which were overlayed to determine location (right column). Top row is an axial slice through the bladder/urinary tract area, second row an axial slice through the knees (arrows) and the third row an axial slice through the liver. A vial containing a known amount of ^19^F-BP was positioned next to the animal for reference.

Preliminary *in vivo* studies were carried out in a 9.4 T scanner with a healthy mouse. We have recently shown that bifunctional BPs accumulate in areas of high bone metabolism such as the end of long bones and bone metastases using SPECT imaging [17], [18]. Hence, we expected **^19^F-BP** to accumulate in bone. However, after intravenous injection, only signals in the bladder/urinary system and liver areas were detected, the former most probably due to renal excretion as expected for a molecule of this size (Fig. 3A) although this cannot be confirmed with the data available. In addition, uptake in other tissues/organs of the same area such as the uterus cannot be ruled out. It is important to note that the ^19^F and ^1^H acquisitions were not performed simultaneously and each modality was acquired with different slice thicknesses (^19^F is 5 times thicker than the ^1^H image), complicating the interpretation of the images. Motion artifacts could also be responsible for the suboptimal overlay of the two modalities. The signal observed in the liver area (Fig. 3C), which is a much bigger organ and hence less affected by these issues (Fig. 3C), is more conclusive to uptake by this organ. Liver uptake is common for lipophilic molecules, and since fluorination is known to increase the lipophilicity of compounds, it is likely to be the result of the trifluoromethyl group. We believe that the lack of bone uptake may be the result of its high lipophilicity, compared to non-fluorinated BPs, resulting in higher liver uptake, and/or fast renal clearance. Indeed, recent reports support the notion that fluorinated groups increase the renal excretion of molecules *in vivo* [22]. Another interesting possibility is that bone binding could have resulted in a chemical shift of the ^19^F resonance that could result in a lack of signal from bone. However, the presence of the expected single resonance in the broad sweep width spectrum performed prior to the imaging session strongly suggests this is not the case.

**Fig. 4:**
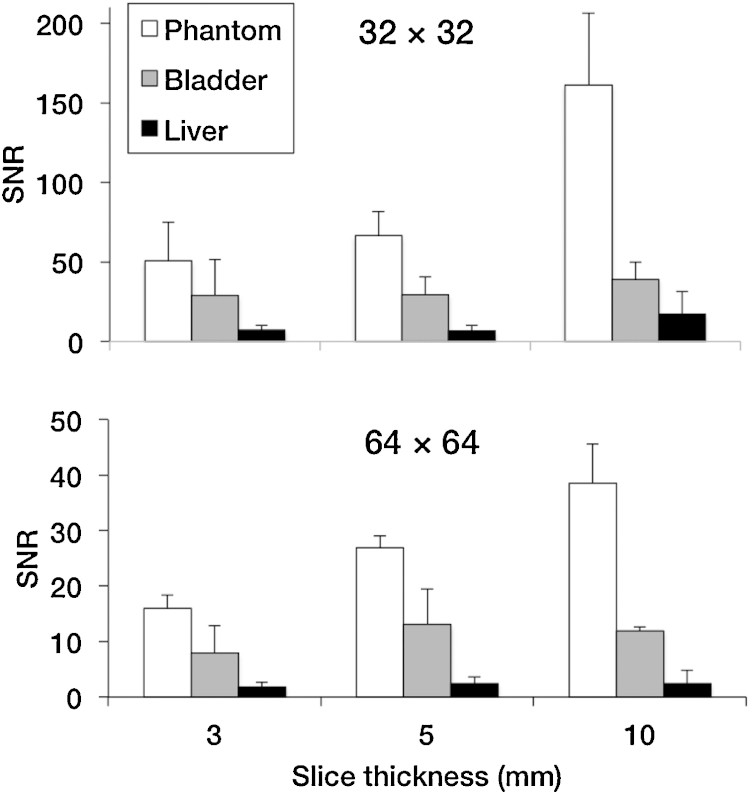
Phantom and animal MR imaging study SNR values. SNR values were calculated for a variety of imaging parameters: matrix size (32 × 32, top; 64 × 64, bottom) and slice thickness (*x*-axis), using ^19^F MRI in the phantom, bladder and liver. Error bars are the result of 3 ROI image analyses.

Another potential reason for the lack of bone uptake observed could be a low signal to noise ratio (SNR). SNR measurements are important in ^19^F-MRI and provide a measure of sensitivity (*i.e.* contrast achieved with amount of imaging agent injected). SNR values of a phantom sample with **^19^F-BP** were found to be in the 50–150 range (32 × 32 matrix size) and 15–40 range (64 × 64 matrix size) for different slice thicknesses. The size of the matrix size is indirectly proportional to the sensitivity, hence the higher values obtained at 32 × 32. For the mouse studies these values were found to be in the 10–40 and 2–12 range, and compare favourably to other animal studies from Bible et al. [23] and Giraudeau et al. [24] (Fig. 4). It is important to note, however, that **^19^F-BP** was found to be toxic at concentrations required to achieve *in vivo* MRI signal (97–119 mM). While other BPs used for nuclear imaging such as ^99m^Tc-MDP are required in micromolar concentrations to obtain image contrast, the amount of BPs required for MRI contrast or therapy is much higher. Toxicity has been observed in animal studies with an amino-bisphosphonate used for therapeutic purposes and injected intravenously (alendronate), at doses of 20 mg/kg. However, doses of 150 mg/kg are required for detecting the ^19^F-MRI signal of ^19^F-BP (for a 20 g mouse). Hence, toxicity is likely to be the result of the bisphosphonate and not the trifluoromethyl group, although further studies are required to confirm this. These results prompted us to abandon the study of **^19^F-BP** for bone imaging and look for potential strategies in order to increase its sensitivity.

### 2.4 Potential strategies to improve sensitivity

The most obvious strategy to improve the sensitivity of **^19^F-BP** is to increase the number of F atoms in the molecule. Interestingly, there are some recent synthetic strategies that would allow us to synthesise a similar BP with several chemically-equivalent F atoms [22]. However, an increase in fluorine content will likely have two main adverse effects. First is solubility, as we anticipate the water solubility will decrease and eventually may result in water-insoluble compounds. The second effect is related to this lower hydrophilicity. We have observed a high degree of liver uptake and hence lipophilicity with a trifluoromethyl group, addition of more fluorine atoms will probably worsen this effect. Another potential adverse effect would be the observed increased *in vivo* rate of excretion of fluorinated agents others and we have observed [22]. A recent proposed method to improve the sensitivity of ^19^F-MRI contrast agents is by positioning the F atoms near a lanthanide in order to enhance their relaxation rates. This technique has been recently explored by Parker and Blamire et al. showing this strategy can result in lower acquisition times and detection limits by as much as 2 orders of magnitude [12], [13], [25]. We hypothesised that, given the known ability of BPs to chelate Ln^3+^ metals and lanthanide oxide materials [19], we could explore this property to enhance the relaxation rate of **^19^F-BP** and hence increase its sensitivity. This method, of course, would not be useful for bone imaging, unless other bifunctional BPs that contain a Ln^3+^ binding group such as a macrocycle chelate (leaving the BP free to bind to bone mineral or inorganic material) between the F-containing motif and the BP are designed. However, it could provide a very useful method to label lanthanide-containing nanomaterials with large numbers of ^19^F atoms and fast acquisition times for other purposes such as cell tracking or molecular imaging using ^19^F-MRI. A preliminary *in vitro* MR study in which we measured the longitudinal and transverse relaxation rates (*R*_1_ and *R*_2_) of **^19^F-BP** in the absence and presence of 1 molar equivalent of different lanthanide salts (Dy^3+^, Er^3+^, Gd^3+^, Ho^3+^ and Tb^3+^) supports the potential of this approach as the presence of Ln^3+^ metals in the solution enhance both relaxation rates by as much as 3 orders of magnitude (supporting information). It is important to note, however, that well-defined and characterised ^19^F-BP-Ln^3+^ complexes would be required in order to validate these findings. We believe this is a strategy that would be particularly useful in conjunction with nanoparticle systems that can combine large numbers of —CF_3_ groups with lanthanide metals at the surface and the required distance from each other. Using this combination, high sensitivity (signal/mole contrast agent) may be achieved thanks to the high numbers of chemically-equivalent ^19^F atoms (hundreds to thousands for a spherical nanometer size particle) with the relaxation capabilities of paramagnetic metals. In addition to the use of paramagnetic ion relaxation, the sensitivity could be further increased in the future by using more efficient MR protocols such as ultrafast sequences recently developed [26].

## 3 Conclusions

**^19^F-BP** was successfully synthesised and characterised. The compound is water soluble and stable and shows a single and narrow fluorine resonance ideally suited for ^19^F-MRI. Phantom studies show that **^19^F-BP** can be imaged using a 9.4 T magnet in the high mM range with SNR ratios similar to other reported probes. An *in vivo* ^19^F-MRI study strongly suggests that ^**19**^**F-BP** was rapidly excreted renally although uptake by other organs/tissue in the area cannot be completely ruled out with our data. Uptake in the liver was also observed which is probably a result of the lipophilicity of the trifluoromethyl group. This data suggests that the lack of bone uptake observed, the natural target of BPs, may be due to the presence of the fluorinated group resulting in fast clearance, as other studies have recently found [22]. More importantly, **^19^F-BP** was found to be toxic at the concentrations used in this study. From these results it is clear that, while **^19^F-BP** may not be useful for bone imaging by itself it may be an useful compound to provide ^19^F signal to many inorganic materials of known affinity towards BPs such as calcium phosphates (*i.e.* hydroxyapatite) and metal oxides, as our recent work suggests. Future work is aimed at using **^19^F-BP** and related BPs to fully exploit this approach.

## 4 Experimental

### 4.1 Materials

Reagents and starting materials were obtained from commercial sources and used as received unless otherwise noted. Organic solvents were of HPLC grade. Water (Type I, 18.2 MΩ cm) was obtained from an ELGA Purelab Option-Q system. Dittmer-Lester’s TLC reagent for the detection of phosphorus was prepared following the original literature protocol [27]. NMR spectra were obtained in a 400 MHz Bruker Avance III (Germany). ^1^H chemical shifts are referenced with respect to the residual solvent peak (*δ*_H_ 4.79 ppm, D_2_O; 7.26 ppm CDCl_3_) [28]. ^31^P resonances were referenced to an external solution of 85% H_3_PO_4_ (*δ*_P_ 0 ppm). ^13^C chemical shifts were referenced to the residual solvent peak (*δ*_C_ 77.16 ppm, CDCl_3_) or left unreferenced (D_2_O). ^19^F resonances were referenced to an external solution of TFA (*δ*_F_ −78.5 ppm). High-resolution mass spectra (HR-MS) were obtained using an Agilent 6500 Accurate-Mass Q-TOF LC–MS system using electrospray ionization. Tetraethyl((dibenzylamino)methylene)bisphosphonate (**1**) and tetraethyl(aminomethylene)bisphosphonate (**2**) were synthesised following published methods [20], [21].

### 4.2 Syntheses

#### 4.2.1 Tetraethyl((2,2,2-trifluoroacetamido)methylene)bisphosphonate. **3**

**2** (200 mg, 0.66 mmol) was dissolved in dry drychloromethane (200 cm^3^) under nitrogen and the flask cooled to 0 °C. After 5 min, trifluoroacetic anhydride (0.274 cm^3^, 1.9 mmol) was added in small portions over 2 min. The ice bath was then removed and the solution was left stirring at room temperature for 2 h during which time the reaction mixture turned slightly yellow. The volatiles were then removed under reduced pressure leaving a clear yellow residue. This residue was dissolved in 2 cm^3^ of dichloromethane and to this mixture were added increasing amounts of a 1% solution of sodium bicarbonate followed by shaking, until the pH of the aqueous layer was 7 (∼10 cm^3^). The organic layer was separated and washed with 3 cm^3^ of water, dried over sodium sulfate, filtered and evaporated under reduced pressure. The residue recrystallised from hexanes after 24 h standing at 4 °C, yielding large quantities of X-ray diffraction-quality crystals (78% yield).

^1^H NMR (CDCl_3_, 400.3 MHz, 298 K) *δ*_H_ (ppm) 4.16 (m, 8H, (—P(O)(OC*H*_2_CH_3_)_2_)), 3.56 (t, ^2^*J*_H-P_ = 22 Hz, 1H, ((P(O)(OEt)_2_)_2_—C*H*—NH—)), 1.34 (s, 12H, (P(O)(OCH_2_C*H*_3_)_2_)); ^13^C NMR (CDCl_3_, 100.7 MHz, 298 K) *δ*_C_ (ppm) 159.1 (q, ^2^*J*_C-F_ = 40 Hz, (—NH—*C*O—CF_3_)), 115.6 (q, *J*_C-F_ = 291 Hz, (—NH—CO*—C*F_3_)), 65.3 (t, ^2^*J*_C-P_ = 3 Hz, (—P(O)(O*C*H_2_CH_3_)_2_)), 43.9 (t, *J*_C-P_ = 148 Hz, ((P(OEt)_3_)_2_—*C*H—NH—)), 16.0 (bs, (—P(O)(OCH_2_*C*H_3_)_2_)); ^31^P{^1^H}-NMR (161.9 MHz, CDCl_3_, 298 K) *δ*_P_ (ppm) 14.02; ^19^F-NMR (376 MHz, CDCl_3_, 298 K) *δ*_F_ (ppm) −76.47; HR-MS (ESI) 400.0939 (M + H^+^, found), 400.0932 (M + H^+^, calculated). 422.0759 (M + Na^+^, found), 422.0721 (M + Na^+^, calculated).

#### 4.2.2 ((2,2,2-trifluoroacetamido)methylene)bisphosphonic acid. **^19^F-BP**

**3** (89 mg, 0.22 mmol) was dissolved in dry drychloromethane (200 cm^3^) under nitrogen and the flask cooled to 0 °C. After 5 min, bromotrimethylsilane (0.444 cm^3^, 3.3 mmol) was added dropwise over 5 min. The ice bath was then removed and the solution was left stirring under nitrogen at room temperature for 24 h during which time the solution turned yellow. The volatiles were then removed under reduced pressure and the residue dissolved in 1.5 cm^3^ of methanol, resulting in a colourless solution. The reaction was left stirring at room temperature for a further 1.5 h followed by evaporation under reduced pressure yielding the product in quantitative yield as a clear sticky oil.

^1^H NMR (D_2_O, 400.3 MHz, 298 K) *δ*_H_ (ppm) 4.60 (t, ^2^*J*_H-P_ = 22 Hz, 1H, ((P(O)(OH)_2_)_2_—C*H*—NH—)); ^13^C NMR (D_2_O, 100.7 MHz, 298 K) *δ*_C_ (ppm) 158.3 (q, ^2^*J*_C-F_ = 40 Hz, (—NH—*C*O—CF_3_)), 115.8 (q, *J*_C-F_ = 285 Hz, (—NH—CO—*C*F_3_)), 47.3 (t, *J*_C-P_ = 136 Hz, ((P(OH)_2_)_2_—*C*H—NH—)); ^31^P{^1^H}-NMR (161.9 MHz, D_2_O, 298 K) *δ*_P_ (ppm) 12.07; ^19^F-NMR (376 MHz, D_2_O, 298 K) *δ*_F_ (ppm) −76.15; HR-MS (ESI) 287.9664 (M + H^+^, found), 287.9650 (M + H^+^, calculated).

### 4.3 X-ray crystallography

Crystal data for 3: C11H22F3NO7P2, *M* = 399.24, triclinic, P-1 (no. 2), *a* = 10.2420(5), *b* = 10.2485(5), *c* = 10.5343(5) Å, *α *= 65.615(4), *β* = 71.735(4), *γ* = 70.761(4)°, *V* = 930.40(9) Å^3^, *Z* = 2, *D*_c_ = 1.425 g cm^−3^, *μ*(Cu-Kα) = 2.700 mm^−1^, *T* = 173 K, colourless blocks, Oxford Diffraction Xcalibur PX Ultra diffractometer; 3670 independent measured reflections (*R*_int_ = 0.0228), *F*^2^ refinement, ^29^*R*_1_(obs) = 0.0352, wR_2_(all) = 0.0972, 3257 independent observed absorption-corrected reflections [|*F*_o_| > 4*σ*(|*F*_o_|), 2*θ*_max_ = 145°], 249 parameters. Crystallographic data for the structures in this paper have been deposited with the Cambridge Crystallographic Data Centre (CCDC ID 960161). Copies of the data can be obtained, free of charge, on application to CCDC, 12 Union Road, Cambridge CB2 1EZ, UK (Fax: +44 1223 336033 or e-mail: deposit@ccdc.cam.ac.uk).

### 4.4 Relaxation rate measurements

The relaxation times *T*_1_ and *T*_2_ of ^19^F in ^19^F-BP and ^19^F-BP + Ln^3+^ mixtures were measured in H_2_O at pH 7 at 400 MHz on a Bruker Avance (Bruker, Ettlingen, Germany) and converted to the *R*_1_ (1/*T*_1_) and R_2_ (1/*T*_2_) rates. *T*_1_ measurements were performed using an inversion recovery technique with 8 inversion times between 0.001 and 4 s, TR = 7 s and 256 averages. *T*_2_ measurements were performed with a spin echo technique with 12 TEs between 0.002 and 0.2 s, TR = 7 s and 8 averages. Analysis was performed using Top Spin software (Bruker, Ettlingen, Germany).

### 4.5 Magnetic resonance imaging (MRI)

#### 4.5.1 Phantom imaging

^19^F-BP at different concentrations (27, 54 and 108 mM) in 250 μL PCR tubes were positioned in a 9.4T Bruker Avance vertical bore scanner using a quadrature volume coil (Bruker, Ettlingen, Germany) alongside a PCR tube containing water. For ^1^H imaging for localisation a RARE sequence was used with TR = 1500 ms, TE = 8.5 ms, NSA = 1, matrix = 256 × 256, FOV = 30 × 30 mm, slc = 1 mm. For the ^19^F imaging the coil was tuned to the ^19^F resonance frequency and a spin echo sequence used with a TR = 3000 ms, TE = 7.6 ms, NSA = 100, matrix = 32 × 32, FOV = 30 × 30 mm, slc = 6 mm, total scan time = 2 h 40 min.

#### 4.5.2 Animal imaging

All animal experiments were performed with licences issued in accordance with the United Kingdom Animals (Scientific Procedures) Act 1986 (UK). One female Balb/c mice (Charles River, Edinburgh, UK), 8–10 weeks old, was anaesthetised using 5% and maintained with 1–2% isoflurane, and injected with 100 μL compound (108 mM in PBS) *via* the tail vein before being transferred to the MRI scanner (9.4T Bruker Avance vertical bore scanner using a quadrature volume coil (Bruker, Ettlingen, Germany)). For ^1^H imaging a FLASH sequence was used with TR = 350 ms, TE = 5.4 ms, FA = 40°, NSA = 5, matrix = 256 × 256, FOV = 30 × 30 mm, slc = 1 mm, 30 slices. For the ^19^F imaging the coil was tuned to the ^19^F resonance frequency and a RARE sequence used with a TR = 1500 ms, TE = 8.5 ms, RARE factor = 4, NSA = 200, matrix = 32 × 32, FOV = 30 × 30 mm, slc = 5 mm, 6 slices, total scan time = 30 min. In addition the same sequence was run, but with FOV = 64 × 64, which had a total scan time of an hour.

^19^F MR images were overlayed on to the ^1^H MR images using ImageJ software (National Institutes of Health, US). To calculate the signal to noise ratios (SNR) of the phantom, bladder and liver ROIs were drawn around the object and also in the background and then values inputted into the following equation taking into account Edelsteins correction factor: SNR = Intensity ROI/(STDEV noise)/√(2-π/2) [29].

## References

[1] Partlow K.C., Chen J., Brant J.A., Neubauer A.M., Meyerrose T.E., Creer M.H., Nolta J.A., Caruthers S.D., Lanza G.M., Wickline S.A. (2007). FASEB J..

[2] Boehm-Sturm P., Mengler L., Wecker S., Hoehn M., Kallur T. (2011). PLoS One.

[3] Bonetto F., Srinivas M., Heerschap A., Mailliard R., Ahrens E.T., Figdor C.G., de Vries I.J.M. (2011). Int. J. Cancer.

[4] Srinivas M., Cruz L.J., Bonetto F., Heerschap A., Figdor C.G., de Vries I.J.M. (2010). Biomaterials.

[5] Srinivas M., Heerschap A., Ahrens E.T., Figdor C.G., de Vries I.J.M. (2010). Trends Biotechnol..

[6] Flögel U., Ding Z., Hardung H., Jander S., Reichmann G., Jacoby C., Schubert R., Schrader J. (2008). Circulation.

[7] Flögel U., Su S., Kreideweiß I., Ding Z., Galbarz L., Fu J., Jacoby C., Witzke O., Schrader J. (2011). Am. J. Transplant..

[8] Southworth R., Kaneda M., Chen J., Zhang L., Zhang H., Yang X., Razavi R., Lanza G., Wickline S.A. (2009). Nanomed. Nanotechnol. Biol. Med..

[9] Lim Y.T., Cho M.Y., Kang J.-H., Noh Y.-W., Cho J.-H., Hong K.S., Chung J.W., Chung B.H. (2010). Biomaterials.

[10] Waters E., Chen J., Allen J., Zhang H., Lanza G., Wickline S., Cardiov J. (2008). Magn. Reson..

[11] Giraudeau C., Geffroy F., Mériaux S., Boumezbeur F., Robert P., Port M., Robic C., Bihan D., Lethimonnier F., Valette J. (2013). Angiogenesis.

[12] Chalmers K.H., Botta M., Parker D. (2011). Dalton Trans..

[13] Chalmers K.H., De Luca E., Hogg N.H.M., Kenwright A.M., Kuprov I., Parker D., Botta M., Wilson J.I., Blamire A.M. (2010). Chem. Eur. J..

[14] Mizukami S., Matsushita H., Takikawa R., Sugihara F., Shirakawa M., Kikuchi K. (2011). Chem. Sci..

[15] Terreno E., Castelli D.D., Viale A., Aime S. (2010). Chem. Rev..

[16] Sandiford L., Phinikaridou A., Protti A., Meszaros L.K., Cui X., Yan Y., Frodsham G., Williamson P.A., Gaddum N., Botnar R.M., Blower P.J., Green M.A., de Rosales R.T.M. (2013). ACS Nano.

[17] de Rosales R.T.M., Finucane C., Foster J., Mather S.J., Blower P.J. (2010). Bioconjug. Chem..

[18] de Rosales R.T.M., Finucane C., Mather S.J., Blower P.J. (2009). Chem. Commun..

[19] de Rosales R.T.M., Tavaré R., Paul R.L., Jauregui-Osoro M., Protti A., Glaria A., Varma G., Szanda I., Blower P.J. (2011). Angew. Chem. Int. Ed..

[20] Kantoci D., Denike J.K., Wechter W.J. (1996). Synth. Commun..

[21] Kubicek V., Rudovsky J., Kotek J., Hermann P., Elst L.V., Muller R.N., Kolar Z.I., Wolterbeek H.T., Peters J.A., Lukes I. (2005). J. Am. Chem. Soc..

[22] Jiang Z.X., Liu X., Jeong E.K., Yu Y.B. (2009). Angew. Chem. Int. Ed..

[23] Bible E., Dell'Acqua F., Solanky B., Balducci A., Crapo P.M., Badylak S.F., Ahrens E.T., Modo M. (2012). Biomaterials.

[24] Giraudeau C., Djemaï B., Ghaly M.A., Boumezbeur F., Mériaux S., Robert P., Port M., Robic C., Bihan D.L., Lethimonnier F., Valette J. (2012). NMR Biomed..

[25] Chalmers K.H., Kenwright A.M., Parker D., Blamire A.M. (2011). Magn. Reson. Med..

[26] Schmid F., Holtke C., Parker D., Faber C. (2013). Magn. Reson. Med..

[27] Ryu E.K., Maccoss M. (1979). J. Lipid Res..

[28] Gottlieb H.E., Kotlyar V., Nudelman A. (1997). J. Org. Chem..

[29] Tofts P.S. (2004). The Measurement Process: MR Data Collection and Image Analysis, Quantitative MRI of the Brain.

