# Supplementary material for: A bisphosphonate for 19F-magnetic resonance imaging
Source: J Fluor Chem. 2016 Apr;184:58–64. doi: 10.1016/j.jfluchem.2016.02.008 (PMC4834630; doi:10.1016/j.jfluchem.2016.02.008)
Supplement: Supplementary file 1 [file mmc1.docx]

**SUPPORTING INFORMATION FOR:**

A Bisphosphonate for ^19^F-Magnetic Resonance Imaging

Gavin D. Kenny^1^, Karen P. Shaw^1^, Saranja Sivachelvam^1^, Andrew J.P. White^2^ and Rafael T. M. de Rosales^1,*^

^1^ Division of Imaging Sciences and Biomedical Engineering, King's College London, St Thomas' Hospital, London, SE1 7EH, UK

^2^ Department of Chemistry, Imperial College London, Exhibition Road, South Kensington, London, SW7 2AZ, UK

**X-ray crystallography**

**The X-ray crystal structure of 3**

The CF_3_ group in the structure of 3 was found to be disordered. Three orientations were identified, of *ca.* 68, 19% and 13% occupancy, their geometries were optimised, the thermal parameters of adjacent atoms were restrained to be similar, and only the atoms of the major occupancy orientation were refined anisotropically (the remainder were refined isotropically).


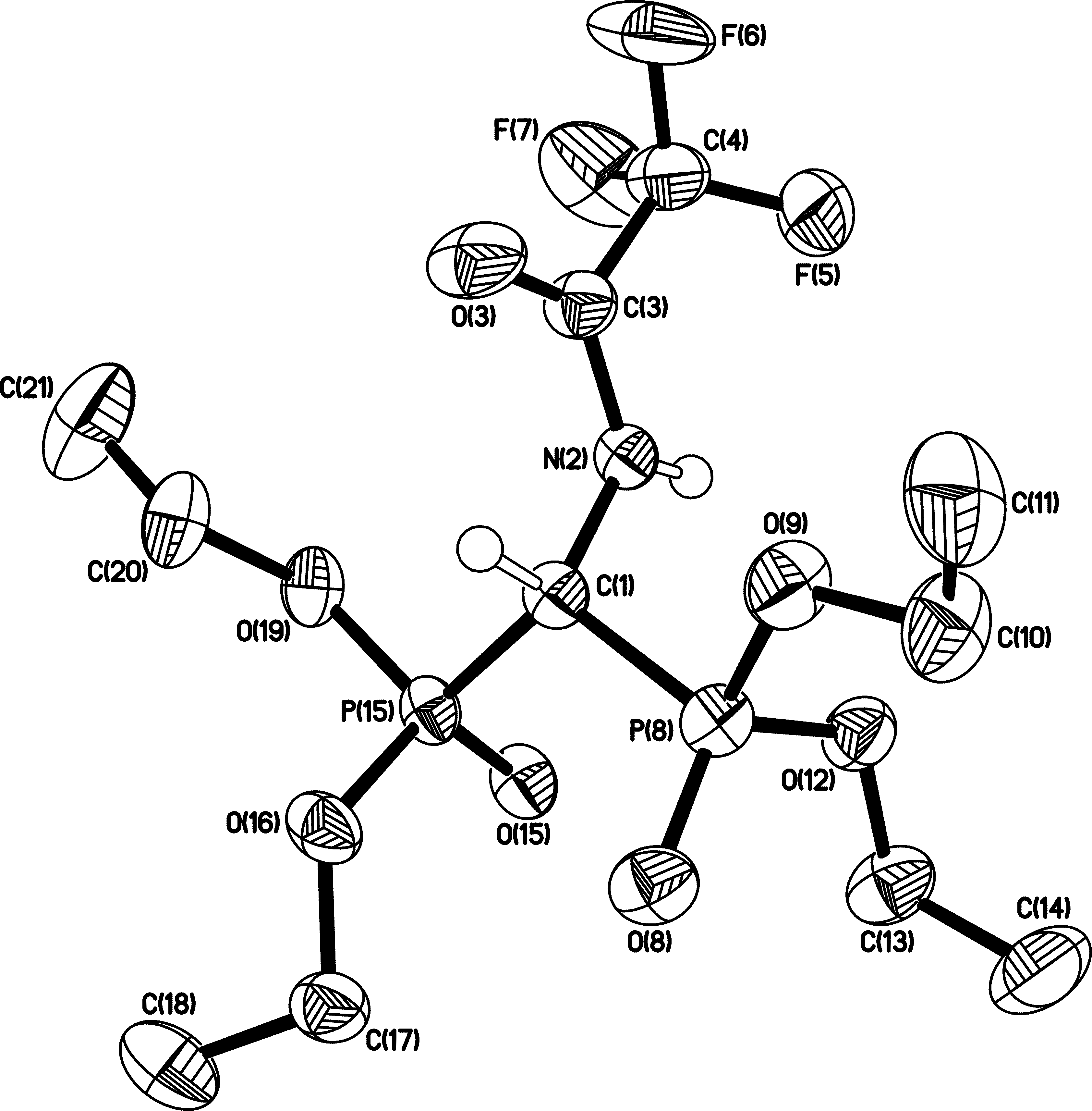


**Fig. S1.** The crystal structure of 3 (50% probability ellipsoids).

**Table S1.** ^19^F-MR relaxation rate measurements at 9.4 T and 25 °C. Please note these measurements were obtained from free ^19^F-BP and in the presence of lanthanide salts (1 equiv), not from single ^19^F-BP-Ln complexes.

|  | *R*_1_ (s^-1^) | *R*_2_ (s^-1^) |
| --- | --- | --- |
| ^19^F-BP | 0.8 | 1.3 |
| ^19^F-BP + Ho^3+^ | 132 | 735 |
| ^19^F-BP + Er^3+^ | Too high | Too high |
| ^19^F-BP + Dy^3+^ | Too high | Too high |
| ^19^F-BP + Tb^3+^ | Too high | Too high |
| ^19^F-BP + Gd^3+^ | Too high | Too high |
