# Supplementary material for: A bisphosphonate for 19F-magnetic resonance imaging
Source: J Fluor Chem. 2016 Apr;184:58–64. doi: 10.1016/j.jfluchem.2016.02.008 (PMC4834630; doi:10.1016/j.jfluchem.2016.02.008)
Supplement: Supplementary file 2 [file mmc2.pdf]

School of Medicine
at Guy's, King's College and St Thomas Hospitals

Division of Imaging Sciences & Biomedical Engineering

Dr. Rafael T. M. de Rosales
Lecturer in Imaging Chemistry
Rayne Institute, 4th Floor, Lambeth Wing
St Thomas' Hospital, London, SE1 7EH
E-Mail: rafael.torres@kcl.ac.uk

KING'S College LONDON
University of London

20th February 2016

Dear Prof. Haufe,

Thanks for your last communication regarding our manuscript "A Bisphosphonate for ^{19}F-Magnetic Resonance Imaging" by Gavin Kenny *et al.* We have carefully considered reviewer 2's comments (please see our reply below) and taken them into account in the text. Below you'll find a list of the changes made in this last version. We hope the manuscript is now acceptable for publication as an article in the *Journal of Fluorine Chemistry*.

Please do not hesitate to contact me if you require any more information.

Yours sincerely,

Dr Rafael T. M. de Rosales

CHANGES TO THE LAST VERSION OF THE MANUSCRIPT:

1) We have removed all text in red font from the previous version.

2) We have changed the format of the references, as suggested by the editor.

3) We have included the following text in page 12 to discuss the injected dose of the probe in relation to that of other bisphosphonates used clinically:

   "While other BPs used for nuclear imaging such as ^{99m}Tc-MDP are required in micromolar concentrations to obtain image contrast, the amount of BPs required for MRI contrast or therapy is much higher. Toxicity has been observed in animal studies with an amino-bisphosphonate used for therapeutic purposes and injected intravenously (alendronate), at doses of 20 mg/kg. However, doses of 150 mg/kg are required for detecting the ^{19}F-MRI signal of ^{19}F-BP (for a 20 g mouse). Hence, toxicity is likely to be the result of the bisphosphonate and not the trifluoromethyl group, although further studies are required to confirm this."
